# Effects of high levels of glucose on the steroidogenesis and the expression of adiponectin receptors in rat ovarian cells

**DOI:** 10.1186/1477-7827-6-11

**Published:** 2008-03-19

**Authors:** Christine Chabrolle, Eric JeanPierre, Lucie Tosca, Christelle Ramé, Joëlle Dupont

**Affiliations:** 1 Unité de Physiologie de la Reproduction et des Comportements, Institut National de la Recherche Agronomique, 37380 Nouzilly, France; 2 Unité d'Endocrinologie, de Diabétologie et des Maladies Métaboliques, CHRU Bretonneau, 37000 Tours, France

## Abstract

**Background:**

Reproductive dysfunction in the diabetic female rat is associated with altered folliculogenesis and steroidogenesis. However, the molecular mechanisms involved in the reduction of steroid production have not been described. Adiponectin is an adipocytokine that has insulin-sensitizing actions including stimulation of glucose uptake in muscle and suppression of glucose production in liver. Adiponectin acts via two receptor isoforms – AdipoR1 and AdipoR2 – that are regulated by hyperglycaemia and hyperinsulinaemia in liver and muscle. We have recently identified AdipoR1 and AdipoR2 in rat ovary. However, their regulation in ovaries of diabetic female rat remains to be elucidated.

**Methods:**

We incubated rat primary granulosa cells in vitro with high concentrations of glucose (5 or 10 g/l) + or - FSH (10-8 M) or IGF-1 (10-8 M), and we studied the ovaries of streptozotocin-induced diabetic rats (STZ) in vivo. The levels of oestradiol and progesterone in culture medium and serum were measured by RIA. We used immunoblotting to assay key steroidogenesis factors (3beta HSD, p450scc, p450 aromatase, StAR), and adiponectin receptors and various elements of signalling pathways (MAPK ERK1/2 and AMPK) in vivo and in vitro. We also determined cell proliferation by [3H] thymidine incorporation.

**Results:**

Glucose (5 or 10 g/l) impaired the in vitro production in rat granulosa cells of both progesterone and oestradiol in the basal state and in response to FSH and IGF-1 without affecting cell proliferation and viability. This was associated with substantial reductions in the amounts of 3beta HSD, p450scc, p450 aromatase and StAR proteins and MAPK ERK1/2 phosphorylation. In contrast, glucose did not affect the abundance of AdipoR1 or AdipoR2 proteins. In vivo, as expected, STZ treatment of rats caused hyperglycaemia and insulin, adiponectin and resistin deficiencies. Plasma progesterone and oestradiol levels were also reduced in STZ rats. However, the amounts of 3beta HSD and p450 aromatase were the same in STZ rat ovary and controls, and the amounts of StAR and p450scc were higher. Streptozotocin treatment did not affect adiponectin receptors in rat ovary but it increased AMPK phosphorylation without affecting MAPK ERK1/2 phosphorylation.

**Conclusion:**

High levels of glucose decrease progesterone and oestradiol production in primary rat granulosa cells and in STZ-treated rats. However, the mechanism that leads to reduced ovarian steroid production seems to be different. Furthermore, adiponectin receptors in ovarian cells are not regulated by glucose.

## Background

Diabetes is associated with increased risk of various diseases including cardiovascular disorders, and is also linked to reproductive problems such as impaired folliculogenesis and steroidogenesis, anovulation, and spontaneous abortions [1-6]. Although several studies have shown altered ovarian function in association with diabetes, the molecular alterations in ovarian steroid metabolism that could explain these reproductive dysfunctions remain to be elucidated. Cholesterol metabolism is profoundly altered in the diabetic condition [7,8], and cholesterol is the essential precursor molecule in the synthesis of steroid hormones. Follicular development and ovulation are dependent on proliferative and differentiation changes in granulosa cells (GCs) and thecal cells, which undergo steroidogenesis upon stimulation with gonadotropins and intraovarian cytokines [9]. For example, FSH (Follicle Stimulating Hormone) and IGF-1 (Insulin-like Growth Factor-1) stimulate the expression of steroidogenic enzymes including the cholesterol side-chain cleavage cytochrome p450 (p450scc), 3β-hydroxysteroid dehydrogenase (3βHSD), cytochrome p450 aromatase (p450 aromatase) and the steroidogenic acute regulatory protein (StAR), which is a protein that participates in the transport of cholesterol from the mitochondrial outer membrane to the inner membrane [10]. The MAPK ERK1/2 signalling pathway has been reported to be involved in the regulation of steroidogenesis in granulosa cells. Indeed, some studies suggest that activation of MAPK ERK1/2 is required for promoting steroidogenesis and steroidogenic gene expression in granulosa cells [11,12]. Furthermore, we have recently shown that the activation of AMPK, a key regulator of cellular energy homeostasis, reduces progesterone secretion through inhibition of the MAPK ERK1/2 signalling pathway in rat and bovine granulosa cells [13,14]. However, it remains to be determined whether diabetes is associated with abnormalities in the abundance of ovarian p450scc, 3βHSD, StAR, p450 aromatase or in MAPK ERK1/2 and AMPK phosphorylations.

Alterations of glucose concentrations can profoundly affect some reproductive process. For example, glucose is required for hormone-induced maturation, but under the appropriate culture conditions, elevated levels of glucose can suppress maturation [15]. However, these effects can be reversed by insulin [15]. Adiponectin is an adipokine that has insulin sensitizing actions including stimulation of glucose uptake in skeletal muscle and suppression of glucose production in liver [16]. Hence, adiponectin has attracted great interest as an antidiabetic agent. Adiponectin acts via two receptor isoforms, AdipoR1 and AdipoR2, which have different tissue distributions [17]. Expression of AdipoR isoforms can be regulated by hyperinsulinaemia and hyperglycaemia [18]. Adiponectin is a key adipokine in the regulation of energy metabolism; however, it is also able to control some reproductive functions. Recently, the adiponectin system (adiponectin, AdipoR1 and AdipoR2) was described in rat and human hypothalamus [19] and pituitary [20]. In rat primary pituitary cells, recombinant adiponectin regulates GnRH receptor expression [20]. In mouse LβT2 gonadotrope cells and in rat pituitary cells, recombinant adiponectin inhibits LH secretion [20,21]. Moreover, adiponectin and AdipoR2 are expressed in rat and human placenta and rat placental adiponectin mRNA increases in abundance during pregnancy whereas AdipoR2 has the opposite pattern [22]. Adiponectin and its receptors are also present in ovary of various species including pig [23,24], chicken [25,26] and rat [27]. Ledoux et al. reported that recombinant adiponectin has a direct effect on gene expression in porcine follicular cells associated with ovarian follicle remodelling [24]. In recent work, our group showed that adiponectin is mainly expressed in rat theca-interstitial cells and could exert paracrine effects on the steroidogenesis of granulosa cells [27]. The expression of the adiponectin system in insulin target tissues (muscle, adipose tissue and liver) in human and animal diabetic models has been studied [27-29]. However, the expression of the adiponectin system in reproductive tissues in diabetic animals has not been explored.

We report an investigation of ovarian steroid production and the protein levels of key factors involved in steroidogenesis in two conditions: in primary rat granulosa cells in the presence of high glucose concentrations; and in streptozotocin-treated female rats. In these conditions, we also assayed adiponectin receptor proteins in ovarian cells.

## Methods

### Hormones and reagents

Purified ovine FSH-20 (oFSH) (lot no.AFP-7028D, 4453 IU/mg, with an FSH activity = 175 times the activity of oFSH-S1) used for culture treatment was a gift from NIDDK, National Hormone Pituitary Program, Bethesda, MD, USA. Recombinant human insulin-like growth factor-I (IGF-I) was from Sigma (St Louis, MO, USA). Recombinant human adiponectin produced in the NSO mammalian cell system was obtained from R&D (Lille, France). Glucose was from VWR (Fontenay sous bois, France).

### Antibodies

Rabbit polyclonal antibodies to adiponectin receptor 2 (4–39) and adiponectin receptor 1 (41–65) were from Phoenix Pharmaceuticals Inc. (Belmont, CA, USA). Rabbit polyclonal antibodies to phospho-ERK1/2 (Thr202/Tyr204), and phospho-AMPK alpha Thr172 were purchased from New England Biolabs Inc (Beverly, MA). Rabbit polyclonal antibodies to AMPKα1 were obtained from Upstate Biotechnology Inc (Lake, Placid, NY, USA). Rabbit polyclonal antibodies to ERK2 (C14) were purchased from Santa Cruz Biotechnology (Santa Cruz, CA). Mouse monoclonal antibody to vinculin was obtained from Sigma (St. Louis, MO, USA). Rabbit polyclonal antibodies against p450scc, StAR and 3βHSD were generously provided by Dr. Dale Buchanan Hales (University of Illinois, Chicago, USA) and Dr. Van Luu-The (CHUL Research Center and Laval University, Canada), respectively. Mouse monoclonal antibody against p450 aromatase was purchased from Serotec (Varilhes, France). All antibodies were used at 1/1000 dilution for western blotting.

### Animals and experimental procedures

All procedures were approved by the Agricultural Agency and the Scientific Research Agency and conducted in accordance with the guidelines for Care and Use of Agricultural Animals in Agricultural Research and Teaching. Eight week-old female Wistar rats (n = 18) were purchased from Janvier Laboratories (France). They were housed with controlled temperature and photoperiod (10 h darkness, 14 h light, light on from 0600–2000 h). The animals had ad libitum access to food and water. Diabetes was induced in one group of rats (n = 12) by an intraperitoneal injection of 1 ml of 50 mM sodium citrate solution (pH 4.5) containing streptozotocin (STZ, 55 mg/kg body weight). Control female rats (C, n = 6) were injected with 50 mM sodium citrate solution (pH 4.5). One week after injection, plasma glucose levels were checked for each animal and diabetes was confirmed (plasma glucose level >3 g/l). Then, control and STZ-treated rats were killed at diestrus stage. One sample of blood was taken and then tissues (ovaries, leg muscles and liver) were removed and frozen for western blotting.

### Isolation and culture of rat granulosa cells

Immature female Wistar rats (21 days) were injected subcutaneously with DES (diethylstilbestrol, 1 mg/day) every day for three days to increase the amount of granulosa cells as previously described [14,30]. On the third day of DES treatment the animals were killed and the ovaries removed aseptically and transferred to culture medium. Granulosa cells were harvested by puncturing the follicles allowing expulsion of the cells. Cells were recovered by centrifugation, washed with fresh medium and counted in a hemocytometer. The culture medium used was McCoy's 5A supplemented with 20 mmol/L Hepes, penicillin (100 U/ml), streptomycin (100 mg/l), L-glutamine (3 mmol/l), 0.1% BSA, 0.1 μmol/l androstenedione, 5 mg/l transferrin, 20 μg/l selenium and 5% FBS. The cells were first cultured for 48 h with no other treatment and then incubated in DMEM medium without glucose and serum but containing penicillin (100 U/ml), streptomycin (100 mg/l), L-glutamine (3 mmol/l) and 0.1 μmol/l androstenedione with or without test reagents (glucose, FSH, IGF-1) for the appropriate time as indicated in the legend of the figures. All cultures were performed under a water-saturated atmosphere of 95% air/5% CO2 at 37°C.

### Western blotting

Lysates of granulosa cells or tissue were prepared on ice with an Ultraturax homogenizer in lysis buffer (10 mM Tris (pH 7.4), 150 mM NaCl, 1 mM EDTA, 1 mM EGTA, 0.5% Igepal) containing various protease inhibitors (2 mM PMSF, 10 mg/ml leupeptin, 10 mg/ml aprotinin) and phosphatase inhibitors [100 mM sodium fluoride, 10 mM sodium pyrophosphate, 2 mM sodium orthovanadate, (Sigma, l'Isle d'Abeau Chesnes, France)]. Lysates were centrifuged at 13,000 g for 20 min at 4°C, and the protein concentration in the supernatants was determined using a colorimetric assay (kit BC Assay, Uptima Interchim, Montluçon, France).

Cell extracts were subjected to electrophoresis on 10% (w:v) SDS-polyacrylamide gel under reducing conditions. The proteins were then electrotransferred onto nitrocellulose membranes (Schleicher and Schuell, Ecquevilly, France) for 2 h. The membranes were incubated for 1 h at room temperature with Tris-buffered saline (TBS, 2 mM Tris-HCl, pH 8.0, 15 mM NaCl, pH 7.6), containing 5% nonfat dry milk powder (NFDMP) and 0.1% Tween-20 to saturate non-specific sites. Then, the membranes were incubated overnight at 4°C with appropriate antibodies (final dilution 1:1,000), in TBS containing 0.1% Tween-20 and 5% NFDMP. They were washed in TBS-0.1% Tween-20, incubated for 2 h at room temperature with a horseradish peroxidase-conjugated anti-rabbit or anti-mouse IgG (final dilution 1:10,000; Diagnostic Pasteur, Marnes-la-Coquette, France) in TBS, 0.1% Tween-20 5% NFDMP, and washed again in TBS-0.1% Tween-20. The signal was detected by ECL (enhanced chemiluminescence, Amersham Pharmacia Biotech, Orsay, France).

The films were analyzed and signals quantified with the software MacBas V2.52 (Fuji PhotoFilm, USA, Inc.). The results are expressed as the signal intensity in arbitrary units after normalization to the signal for vinculin, used as an internal standard, and each value reported corresponds to the means of three independent experiments.

### Thymidine incorporation into granulosa cells

Granulosa cells (2 × 10^5 ^viable cells/500 μl) were cultured in 24-well dishes in McCoy's 5A medium containing 10% FBS for 48 h and were then serum starved for 24 h in DMEM medium without glucose: then 1 μCi/μl of [3H] thymidine (Amersham Life Science, Arlington Heights, IL) was added in the presence or absence of various concentrations of glucose and/or FSH (10^-8^M) and IGF-1 (10^-8 ^M). Cultures were maintained at 37°C under 5% CO_2 _in air. After 24 h of culture, excess of thymidine was removed by washing twice with phosphate-buffered saline (PBS), and the samples were fixed with cold trichloroacetic acid 50% for 15 min and lysed by addition of 0.5 N NaOH. The radioactivity was determined by addition of scintillation fluid (Packard Bioscience) and counting in a β-photomultiplier.

### Progesterone and oestradiol radioimmunoassay

The concentration of progesterone (P) and oestradiol (E2) in the culture medium of granulosa cells was measured after 48 h of culture by a radioimmunoassay protocol as previously described [31-33] and adapted to measure steroids in cell culture media. The limit of detection of P was 12 pg/tube (60 pg/well) and the intra- and interassay coefficients of variation were less than 10% and 11%, respectively. The limit of detection of E2 was 1.5 pg/tube (7.5 pg/well) and the intra-and interassay coefficients of variation were less than 7% and 9%, respectively. Results are expressed as the amount of steroids secreted per 48 h per 100 μg of protein, and values reported are means ± SE of three cultures of granulosa cells. For each culture, about 30 to 40 rats were used and each condition (FSH, IGF-1, glucose alone or combined with IGF-1 or FSH) was analyzed four times independently.

After extraction of steroids from serum, the concentration of progesterone was measured by the RIA method as previously described [34]. The oestradiol concentration in the serum was measured with a RIA KIT (DIASORIN, Antony, France).

### Glucose, adiponectin, resistin and insulin plasma levels

Plasma glucose was assayed by the glucose oxidase method (Glucose Beckman Analyser 2, Beckman, Palo Alto, CA). Plasma adiponectin and resistin were assayed with a rat adiponectin ELISA kit (Phoenix Pharmaceuticals, Inc, Belmont, CA, USA) and a rat resistin ELISA kit (BioVendor laboratory Medicine, Inc, Heidelberg, Germany), respectively. Serum insulin levels were determined using a rat insulin ELISA kit (Mercodia AB, Uppsala, Sweden).

### Statistical analysis

All experimental data are presented as the mean ± SE. One t-test (for comparison of two means, Control and STZ) or one-way analysis of variance (ANOVA) (for comparison of various means) were used to test differences; if ANOVA revealed significant effects, the means were compared by Newman's test, with P < 0.05 considered significant.

## Results

### Effect of glucose on basal and FSH- or IGF-1-stimulated oestradiol and progesterone production in rat granulosa cells

**Figure 1:**
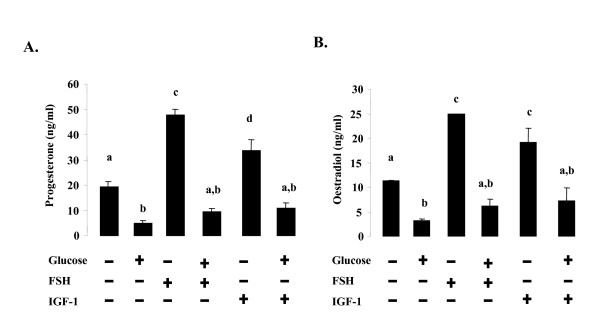
**Effect of glucose treatment on basal and FSH- or IGF-1-stimulated progesterone and oestradiol secretions by rat granulosa cells**. Granulosa cells from immature rats were cultured for 48 h in medium with serum and then in serum-free medium in the absence or in the presence of 10 g/l glucose ± 10^-8 ^M FSH or 10^-8 ^M IGF-1 for another 48 h (**A **and **B**) as described in Materials and Methods. The culture medium was then collected and assayed for progesterone (**A**) and oestradiol (**B**) by RIA. Results are means ± SE for three groups of granulosa cells. Each group of granulosa cells was obtained from about 30 rats. Bars with different letters are significantly different (p < 0.05). The letter "a" indicates values which are not significantly different from control (without FSH or IGF-1 and glucose).

The effects of glucose treatment on steroidogenesis in rat granulosa cells were first examined in incubations containing various concentrations of glucose for 48 h. Concentrations of glucose greater than 5 g/l were found to inhibit the syntheses of progesterone and oestradiol (data not shown). We also determined whether glucose (10 g/l) affected the production of progesterone and oestradiol in response to FSH or IGF-1. In the presence of FSH (10^-8^M), glucose (10 g/l, 48 h) decreased the secretion of progesterone (p < 0.01) and oestradiol (p < 0.01) by a factor of about five (Fig. 1A and 1B). In the presence of IGF-1 (10^-8^M), glucose (10 g/l, 48 h) decreased the secretion progesterone (p < 0.01) and oestradiol (p < 0.01) by a factor of about three (Fig. 1A and 1B). Similar results were obtained with a lower dose of glucose (5 g/l, data not shown). Thus, a high glucose concentration (5 or 10 g/l) decreased both basal and FSH-or IGF-1-stimulated progesterone and oestradiol production in rat granulosa cells.

**Figure 2:**
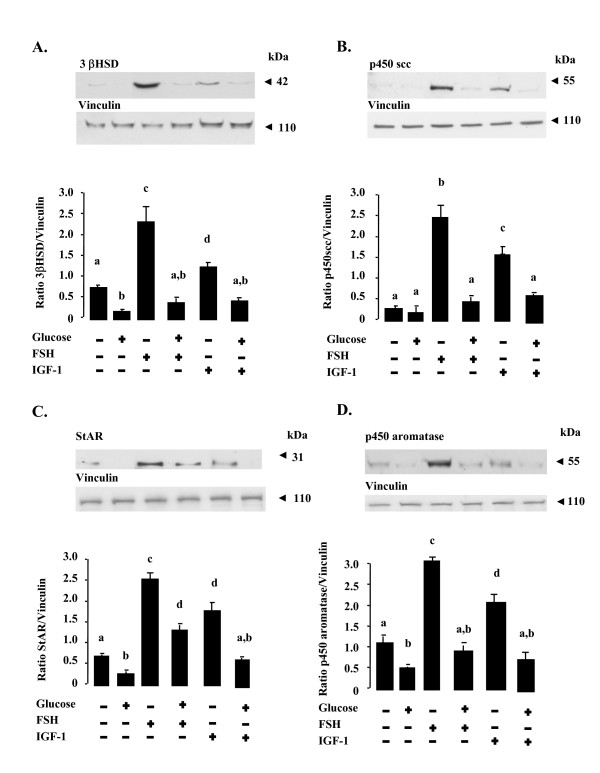
**Effect of glucose treatment on the amounts of the 3βHSD, p450scc, StAR and p450 aromatase proteins in rat granulosa cells**. Protein extracts from rat granulosa cells cultured for 48 h in the absence or in the presence of 10 g/l glucose ± 10^-8 ^M FSH or 10^-8 ^M IGF-1 were subjected to SDS-PAGE as described in Materials and Methods. The membranes were incubated with antibodies raised against the 3βHSD (**A**), p450scc (**B**), StAR (**C**) and p450 aromatase (**D**) proteins. Equal protein loading was verified by reprobing membranes with an anti-vinculin antibody. Results are representative of at least three independent experiments. Blots were quantified and the 3βHSD, p450scc, StAR and p450 aromatase to Vinculin ratios are shown. The results are expressed as means ± SE. Bars with different letters are significantly different (p < 0.05). The letter "a" indicates values which are not significantly different from control (without FSH or IGF-1 and glucose).

We then investigated whether this inhibitory effect of glucose on the production of both progesterone and oestradiol resulted from the production of smaller amounts of the three key enzymes in steroidogenesis (3βHSD, p450scc and p450 aromatase) and/or of StAR, a major cholesterol carrier. Glucose treatment (10 g/l, 48 h) in the presence of FSH (10^-8^M) decreased production of 3βHSD (Fig. 2A, p < 0.001) and p450scc (Fig. 2B, p < 0.001) by a factor of about seven, halved the production of StAR (Fig. 2C, 0.05) and reduced by three-fold the production of p450 aromatase (Fig. 2D, p < 0.001), relative to the values in the presence of FSH without glucose. In the presence of IGF-1 (10^-8^M), glucose decreased the amounts of the three key enzymes in steroidogenesis and StAR by a factor of three relative to IGF-1 treatment without glucose (Fig. 2A to D, p < 0.05). Similar results were obtained with a lower glucose concentration (5 g/l, data not shown).

In the basal state (no FSH or IGF-1), glucose treatment (10 g/l, 48 h) only halved the production of 3βHSD, StAR and p450 aromatase (Fig. 2A, C and 2D, p < 0.05) but did not affect the amount of p450scc protein (Fig. 2B). Thus, the decrease in FSH- or IGF-1-induced progesterone and oestradiol secretions in response to glucose treatment appears to be caused by a reduction in the amounts of the 3βHSD, p450scc, StAR and p450 aromatase proteins. The inhibition of basal progesterone and oestradiol secretions in response to glucose could be the result of a reduction in production of the 3βHSD, StAR and p450 aromatase proteins.

### Effects of glucose on granulosa cell proliferation and viability

We investigated whether the dose of glucose used (5 or 10 g/l) affected the number of granulosa cells in culture, either by induction of mitosis or by altering the cell viability. [^3^H]-thymidine incorporation by granulosa cells treated with 10 g/l glucose was determined after 24 h of culture in the presence or in the absence of FSH (10^-8 ^M) or IGF-1 (10^-8 ^M). As expected, FSH and IGF-1 treatment significantly increased [^3^H]-thymidine incorporation (data not shown). However, glucose treatment (either 5 or 10 g/l) did not affect cell proliferation or cell number (data not shown). Glucose (5 or 10 g/l) had no effect on the cell viability in the absence or in the presence of FSH and IGF-1 as assessed by staining with Trypan blue (data not shown).

### Effects of glucose treatment on the MAPK ERK1/2 and AMPK phosphorylation and on the adiponectin receptor expression in rat granulosa cells

**Figure 3:**
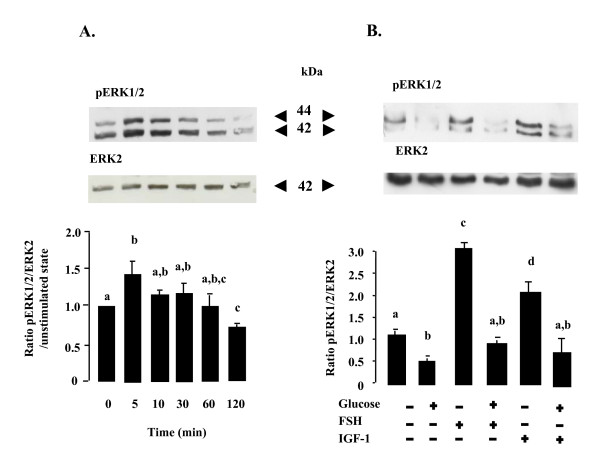
**Effect of glucose treatment on MAPK ERK1/2 phosphorylation in rat granulosa cells**. Granulosa cell lysates were prepared from cells incubated with 10 g/l glucose for various times: 0, 5, 10, 30, 60 or 120 min (A) or with 10 g/l glucose for 48 h in the presence or absence of FSH (10^-8^M) or IGF-1 (10^-8^M). Lysates (50 μg) were resolved by SDS-PAGE, transferred to nitrocellulose membrane, and probed with anti-phospho-MAPK ERK1/2 and then with anti-ERK2 antibodies. Representative blots from three independent experiments are shown. Blots were quantified and the phosphorylated MAPK ERK1/2/ERK2 protein ratio is shown. The results are represented as means ± SE. Bars with different letters are significantly different (p < 0.05). The letter "a" indicates values which are not significantly different from control (without FSH or IGF-1 and glucose).

We examined whether the inhibitory effect of glucose on progesterone and oestradiol production was associated with a variation in the phosphorylation of MAPK ERK1/2 and AMPK. These kinases have been implicated in the regulation of steroidogenesis [11,13]. We analysed the pattern of MAPK ERK1/2 phosphorylation in cells incubated with 10 g/l glucose for various times (0, 5, 10, 30, 60, 120 min, Fig. 3A), or with 10 g/l glucose for 48 h in the presence or absence of FSH (10^-8^M) or IGF-1 (10^-8^M, Fig. 3B). MAPK ERK1/2 phosphorylation was significantly reduced after 120 min of treatment with 10 g/l glucose (p < 0.05, Fig. 3A). It was also decreased in the basal and FSH-or IGF-1-stimulated conditions used for assaying progesterone and oestradiol production (48 h of treatment with 10 g/l glucose in serum-free medium, p < 0.05, Fig. 3B). Similar results were obtained with a lower glucose concentration (5 g/l, data not shown). We also determined phosphorylation of Thr172 in AMPK in similar conditions (effect of glucose in the short and long term); glucose (5 or 10 g/l) had no effect on AMPK phosphorylation (data not shown). We also investigated the effect of a high concentration of glucose (10 g/l) on the amounts of AdipoR1 and AdipoR2 (the adiponectin receptors) in rat granulosa cells and we detected no significant effect (data not shown).

### Glucose, insulin, progesterone and oestradiol plasma levels in control and STZ-treated rats

**Figure 4:**
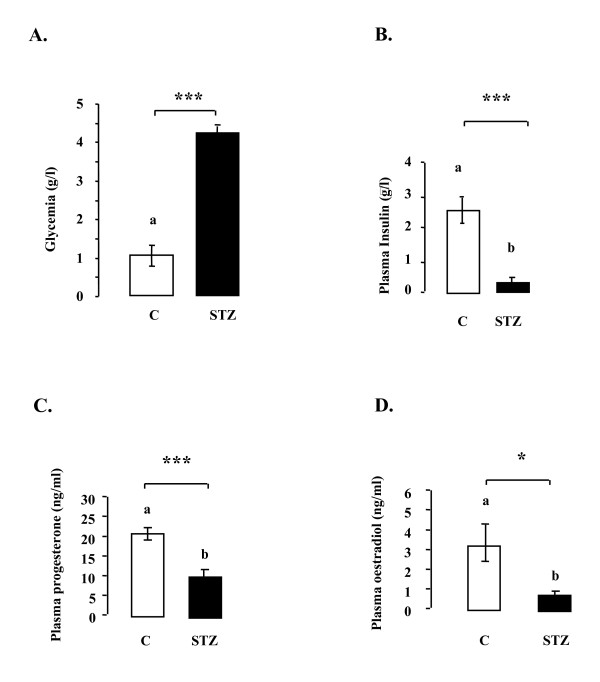
**Plasma glucose (A), insulin (B), progesterone (C) and oestradiol (D) concentrations in the control (n = 6) and STZ-treated rats (n = 12)**. Plasma glucose was assayed by the glucose oxidase method. Plasma insulin concentrations were determined by ELISA and plasma progesterone and oestradiol were assayed by RIA. C: control; STZ: streptozotocin. *p < 0.05, ***p < 0.001

We next studied the steroid production in vivo in streptozotocin-induced diabetic mature female rats. The body weight and plasma glucose levels of control and STZ-treated rats are shown in Table 1. STZ treatment did not alter the body weight whereas it significantly increased the plasma glucose concentrations (glycaemia > 4 g/l, p < 0.0001) to higher than that in control rats (Table 1, Fig. 4A). As expected, serum insulin levels were much lower in STZ-treated rats than in control rats (Fig. 4B, p < 0.001). Progesterone (Fig. 4C, p < 0.001) and oestradiol (Fig. 4D, p < 0.05) concentrations in plasma were also significantly lower in STZ-treated than control animals.

**Table 1:** Body weight and plasma glucose levels of rats before and after streptozotocin treatment.

	Before treatment		After treatment	
	Body weight (g)	Plasma Glucose (g/l)	Body weight (g)	Plasma Glucose (g/l)

Control	179 ± 4	1.20 ± 0.06	230 ± 5	1.1 ± 0.05^a^
Streptozotocin	173 ± 5	1.10 ± 0.02	215 ± 6	4.1 ± 0.11^b^

p < 0.05, different letters indicate significant difference between control and streptozotocin animals.

### 3βHSD, p450scc, StAR and p450 aromatase in control and STZ-treated rat ovaries

**Figure 5:**
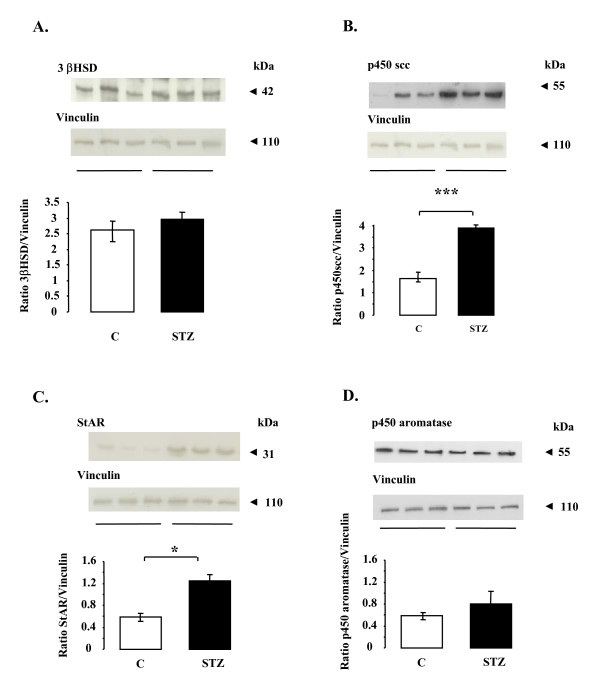
**Effect of STZ treatment on the amounts of the 3βHSD, p450scc, StAR and p450 aromatase proteins in rat ovarian cells**. Protein extracts of ovaries from control and STZ-treated rats were subjected to SDS-PAGE as described in Materials and Methods. The membranes were incubated with antibodies raised against the 3βHSD (**A**), p450scc (**B**), StAR (**C**) and p450 aromatase (**D**) proteins. Equal protein loading was verified by reprobing membrane with an anti-Vinculin antibody. Blots were quantified and the 3βHSD, p450scc, StAR and p450 aromatase/Vinculin ratios are shown. Data are shown as means ± SE, with n = 6 for the control group and n = 12 for the STZ-treated group. *p < 0.05, *p < 0.001.

To examine possible alterations in the key proteins required for progesterone and oestradiol production, we used western-blot analysis of ovarian tissue. There was no significant difference in the amounts of 3βHSD or p450 aromatase in control and STZ-treated rats (Fig. 5A and 5D). In contrast, there was significantly more p450scc (p < 0.001) and StAR (p < 0.05) in STZ-treated animals than in control rats (Fig. 5B and 5C).

### Effect of STZ treatment on the plasma levels of adiponectin and resistin (A) and on the amounts of AdipoR1 and AdipoR2 in rat ovary (B), muscle (C) and liver (D)

**Figure 6:**
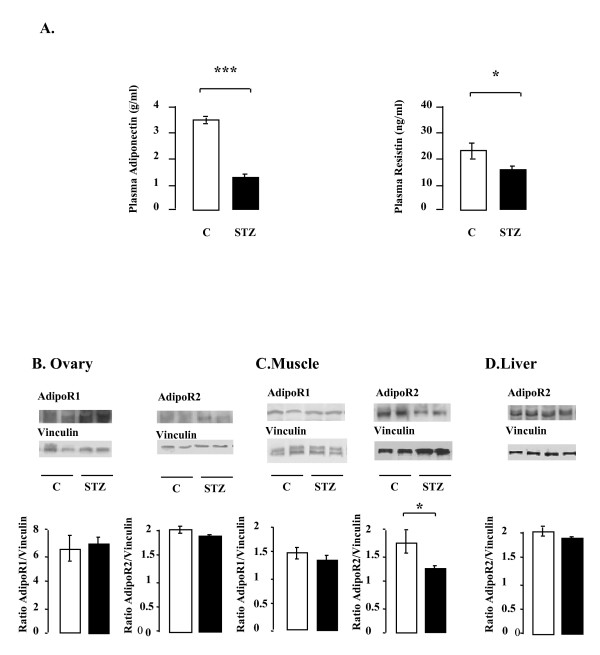
**Effect of STZ treatment on the plasma levels of adiponectin and resistin (A) and on the protein levels of AdipoR1 and AdipoR2 in rat ovary (B), muscle (C) and liver (D)**. **A.A**. Plasma adiponectin and resistin concentrations were determined by ELISA. C: control; STZ: streptozotocin. *p < 0.05, ***p < 0.001. **B, C and D**. Lysates (50 μg) of ovary (B), muscle (C) and liver (D) from control and STZ-treated rats were resolved by SDS-PAGE, transferred to nitrocellulose membrane, and probed with anti-AdipoR1 or anti-AdipoR2 antibodies. Equal protein loading was verified by reprobing the membrane with an anti-Vinculin antibody. Blots were quantified and the AdipoR1, AdipoR2/vinculin ratios are shown. Data are shown as means ± SE, with n = 6 for the control group and n = 12 for the STZ-treated group. *p < 0.05, *p < 0.001.

The concentrations of plasma adiponectin and resistin were significantly lower in STZ-treated rats than in control rats (Fig. 6A). We next assayed the adiponectin receptors in ovary and, as controls in muscle and liver, from control and STZ-treated rats. The amounts of AdipoR1 and AdipoR2 were similar in the ovaries of control and STZ-treated rats (Fig. 6B). STZ treatment did not alter AdipoR1 protein abundance in muscle, but it decreased that of AdipoR2 (p < 0.1, Fig. 6C). AdipoR2 is the only receptor detectable in liver, and was unaffected in this tissue by STZ treatment (Fig. 6D).

### Effects of STZ treatment on the MAPK ERK1/2 and AMPK phosphorylation in rat ovary

**Figure 7:**
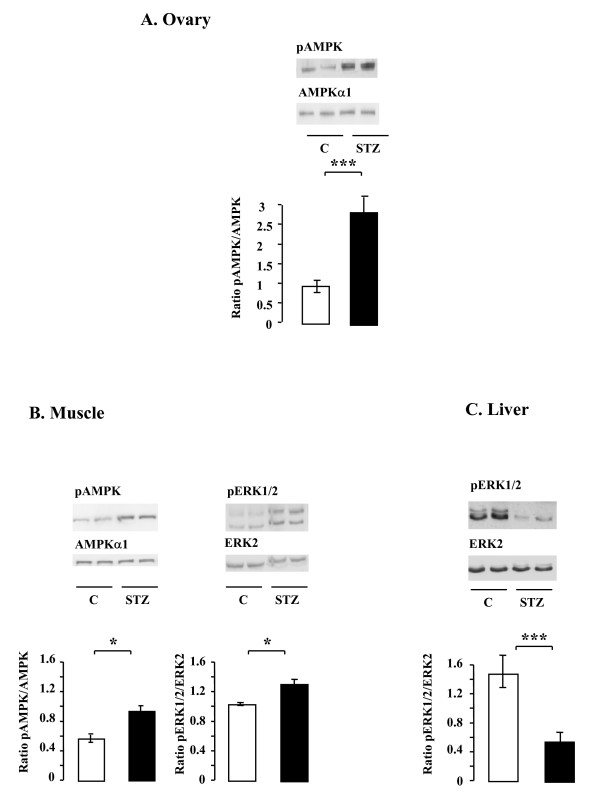
**Effect of STZ treatment on the phosphorylation levels of AMPK and MAPK ERK1/2 in rat ovary (A), muscle (B) and liver (C)**. Lysates (50 μg) of ovary, muscle and liver from control and STZ-treated rats were resolved by SDS-PAGE, transferred to nitrocellulose membrane, and probed with anti-phospho-AMPK, phospho-MAPK ERK1/2 and then with anti-AMPKα1 and anti-ERK2 antibodies, respectively. Blots were quantified and the phosphorylated protein/total protein ratio is shown. Data are shown as means ± SE, with n = 6 for the control group and n = 12 for the STZ-treated group. *p < 0.05, *p < 0.001.

We also analyzed the activation of various signalling pathways, including MAPK ERK1/2 and AMPK, involved in steroidogenesis in rat granulosa cells [11,13]. In ovary, AMPK phosphorylation was increased by STZ treatment (Fig. 7A, p < 0.001) whereas the treatment had no effect on MAPK ERK1/2 phosphorylation (data not shown). In muscle, phosphorylation of both AMPK and MAPK ERK 1/2 were increased by STZ treatment (p < 0.05, Fig. 7B). In contrast, MAPK ERK 1/2 phosphorylation in the liver was strongly reduced by STZ treatment (p < 0.001, Fig. 7C) whereas AMPK phosphorylation was unchanged (data not shown).

## Discussion

We investigated the impact of hyperglycaemia on steroid production by rat ovarian cells. We report that high concentrations of glucose (5 or 10 g/l) decreased both progesterone and oestradiol production by rat primary granulosa cells in vitro, in both the basal state and in response to FSH or IGF-1. This inhibitory effect was associated with a reduction in the amounts of 3βHSD, p450scc, StAR and p450 aromatase and of MAPK ERK1/2 phosphorylation. In contrast, AMPK phosphorylation was not affected by high levels of glucose. We studied the steroidogenesis in vivo, in ovaries of the STZ-treated rats. The streptozotocin treatment significantly decreased plasma concentrations of progesterone and oestradiol. Curiously, this was associated with an increase in the amounts of p450scc and StAR proteins in the ovaries of STZ-treated rats whereas the amounts of 3βHSD and p450 aromatase proteins were similar in STZ-treated and control rats. Furthermore, AMPK phosphorylation was increased in STZ-treated rat ovary whereas the streptozotocin treatment had no effect on MAPK ERK1/2 phosphorylation. We also observed that the abundance of adiponectin receptors was not affected by supra-physiological levels of glucose either in vitro in rat granulosa cells or in vivo in ovaries of STZ-treated rats. Thus, glucose seems to be involved in a series of metabolic pathways that collectively contribute to normal ovarian steroidogenesis.

In the present study, we have shown that glucose treatment decreases basal and FSH-or IGF-1-stimulated steroid production in vitro in rat granulosa cells. Furthermore, this result was associated to a reduction in MAPK ERK1/2 phosphorylation. However, the dose of glucose used in our study is high (5 or 10 g/l), corresponding to supra-physiological levels. There are conflicting reports concerning the involvement of MAPK ERK1/2 in the regulation of steroidogenesis in various steroid-producing cells, probably because different species (rodents and humans) and different culture systems have been used in various studies. Consistent with our findings presented here, studies in rats using primary cultures of granulosa cells demonstrate that inhibition of MAPK ERK1/2 decreased FSH-induced progesterone secretion [14,30,35], 3βHSD [14] and StAR expression [12,36]. MAPK ERK1/2 regulates target gene expression by activating downstream transcription factors including the steroidogenic factor 1 (SF-1) [36]. Furthermore, the 3βHSD type 2 promoter contains a consensus sequence for SF-1 [37]. Thus, high concentrations of glucose (5 or 10 g/l) like those we used may reduce progesterone secretion in rat granulosa cells through inhibition of MAPK ERK1/2 leading to a reduction in the 3βHSD and/or StAR levels and consequently progesterone secretion. We can not also exclude that high concentration of glucose may cause energy stress for the cells and consequently a reduction of the steroid production. However, we observed that high concentrations of glucose did not affect AMPK activation. AMPK is considered to be a master switch in regulating glucose metabolism: it acts as a fuel gauge, being activated in conditions of extreme phosphate depletion and inhibited by high levels of ATP [38]. In granulosa cells, glucose can be metabolized through the pentose phosphate pathway and this leads to the production of ATP [39]. Thus, high levels of glucose should reduce AMPK phosphorylation. However, we can not exclude the possibility that glucose regulates AMPK phosphorylation in rat granulosa cells but only in conditions different to those we used (different doses or after different periods of time). We recently showed that AMPK activation decreases progesterone secretion through MAPK ERK1/2 inhibition in rat granulosa cells. As 10 g/l glucose did not affect AMPK phosphorylation, it probably acts through another molecular mechanism to inhibit MAPK ERK1/2 phosphorylation. There is evidence that inhibition of MAPK ERK1/2 leads not only to a decrease in progesterone secretion but also an increase in the p450 aromatase expression and oestradiol production [12]. We report that 5 or 10 g/l glucose decreased progesterone and oestradiol production, and it is therefore likely that glucose uses a molecular mechanism other than the inhibition of MAPK ERK1/2 to reduce oestradiol production. We found that 5 or 10 g/l glucose did not affect granulosa cell proliferation in the basal state or in response to FSH or IGF-1. These observations are opposite to findings for other cell types. For example, Turner and Bierman [40] and Hehenberger and Hansson [41] have stated that glucose was important for cell proliferation; they report that increasing glucose levels to 18 and 15.5 mM (about 3 g/l), respectively, increases fibroblast proliferation, whereas further increases lead to an inhibition of proliferation [40,41]. Thus, the effect of glucose on cell proliferation seems to depend on the concentration used and the cell type. We have shown that high concentrations of glucose (10 g/l) did not affect the abundance of adiponectin receptors in rat granulosa cells. However, it is plausible that glucose modulates the signalling pathways activated by adiponectin. We recently observed that human recombinant adiponectin activated several signalling pathways including AMPK, MAPK ERK1/2 and p38, and also Akt [27]. Thus, the effects of high glucose levels on the adiponectin response in rat granulosa cells need to be tested.

We also investigated the effect of hyperglycaemia (about 4 g/l or 22 mM glucose) in vivo in the ovary of streptozotocin-induced diabetic rats. As expected, the plasma concentrations of insulin were low in these STZ diabetic rats and they also had lower plasma adiponectin and resistin levels than rat controls. The concentration of adiponectin in plasma is diminished in type 2 diabetes [42] whereas it has been reported that the adiponectin concentration increased in type 1 diabetic patients [43,44]. Our work suggests that STZ rats may have a degree of insulin resistance. We did not analyze the total body fat content. However, when we removed liver and muscle from STZ and Control rats, STZ rats seemed to lose fat mass (data not shown) despite their body weight not being significantly different to controls (mean: 229 g versus 214 g p = 0.1). The decreased fat mass may explain the diminished adiponectin and resistin levels. We observed that plasma progesterone and oestradiol concentrations were lower in STZ-treated rats than in controls suggesting that ovarian steroidogenesis function was altered. The decline in steroid production in several diabetic states is well documented [2,3,45,46]. However, the mechanism of the reduction in ovarian steroidogenesis is not clear. In the present study, no decrease in the amounts of 3βHSD and p450 aromatase was observed whereas the levels of StAR and p450scc proteins were increased in the ovaries of STZ-treated animals. These data are in a good agreement with other studies that report no alteration to p450scc or 3βHSD [47]. Possibly, the activity of these key steroidogenesis enzymes is decreased in STZ-treated rats and this could explain the decline in steroid production in these animals. We found that the protein level of adiponectin receptors (AdipoR1/R2) was similar both in ovaries and in the liver of control and STZ-treated rats. Only the AdipoR2 protein was slightly less abundant in muscle of STZ-treated than control rats. Several studies have explored the mRNA for adiponectin receptors in diabetic human and animal tissues, and the results are the subject of debate. They depend on the body mass index, the level of insulin resistance and especially the tissue type. In human and rodent type 2 diabetic model, mRNAs for insulin and the AdipoR1/R2 are altered in muscle and liver [18,48]. Another group concluded that the adiponectin level is inversely correlated with obesity, diabetes and insulin resistance, whereas the amounts of adipoR1/R2 mRNA increased in muscle in a compensatory effect [28]. In contrast, Hammarstedt's group and Yao's group reported no change in the expression of the two adiponectin receptors in type 2 diabetic patients and rodents [29,49]. In the present study, we observed higher levels of AMPK phosphorylation in the ovary and also in muscle of STZ-treated than control rats. AMPK is activated by energy stress, for example glucose deprivation [50]. In our model, STZ treatment causes a lack of insulin because of the pancreatic function impairment. Thus, hyperglycaemia develops but no glucose can enter into the cell of the insulin target tissues. This cellular stress may involve AMPK activation in the various tissues explored including the ovary despite it not being considered to be a major insulin-dependent tissue. The increase in AMPK activation in the ovary of STZ-treated rats may contribute to the decrease in progesterone secretion in these animals. Indeed, we have recently shown that AMPK activation decreases progesterone secretion in rat granulosa cells [14].

## Conclusion

Our various results suggest that high levels of glucose (5 or 10 g/l) decrease steroid production. However, the mechanisms involved in the reduction in ovarian steroidogenesis depend on the model used (in vitro primary rat granulosa cells or in vivo ovary of STZ-treated rat). In rat granulosa cells, high levels of glucose decrease the protein levels of the main steroidogenesis factors whereas the amounts of these factors are not affected or even increased in the ovaries of STZ-treated rats. Furthermore, high levels of glucose did not affect the abundance of adiponectin receptors in vitro in rat granulosa cells or in vivo in the ovaries of STZ-treated rats.

## Competing interests

The author(s) declare that they have no competing interests.

## Authors' contributions

CC participated together with JD in the design of the study. The experiments were carried out by CC, EJP, CR and LT. Data analysis was performed by CC, EJP, CR, LT and JD. The manuscript was written by CC. All authors have read and approved the final manuscript.
